# The role of the Toll like receptor 4 signaling in sex-specific persistency of depression-like behavior in response to chronic stress

**DOI:** 10.1016/j.bbi.2023.10.006

**Published:** 2023-10-12

**Authors:** Eun-Jeong Yang, Tal Frolinger, Umar Iqbal, Molly Estill, Li Shen, Kyle J. Trageser, Giulio M. Pasinetti

**Affiliations:** a Department of Neurology, Icahn School of Medicine at Mount Sinai, New York, New York 10029, United States; b Department of Neuroscience, Icahn School of Medicine at Mount Sinai, New York, New York 10029, United States; c Geriatric Research, Education and Clinical Center, James J. Peters Veterans Affairs Medical Center, Bronx, New York 10468, United States

**Keywords:** Depression, Chronic stress, Sex difference, Microglia activation, TLR4, Neuroinflammation

## Abstract

Chronic stress is a major risk factor for Major Depressive Disorder (MDD), and it has been shown to impact the immune system and cause microglia activation in the medial prefrontal cortex (mPFC) involved in the pathogenesis of depression. The aim of this study is to further investigate cellular and molecular mechanisms underlying persistent depression behavior in sex specific manner, which is observed clinically. Here, we report that both male and female mice exhibited depression-like behavior following exposure to chronic stress. However, only female mice showed persistent depression-like behavior, which was associated with microglia activation in mPFC, characterized by distinctive alterations in the phenotype of microglia. Given these findings, to further investigate the underlying molecular mechanisms associated with persistent depression-like behavior and microglia activation in female mice, we used translating-ribosome affinity purification (TRAP). We find that Toll like receptor 4 (TLR4) signaling is casually related to persistent depression-like behavior in female mice. This is supported by the evidence that the fact that genetic ablation of TLR4 expression in microglia significantly reduced the persistent depression-like behavior to baseline levels in female mice. This study tentatively supports the hypothesis that the TLR4 signaling in microglia may be responsible for the sex differences in persistent depression-like behavior in female.

## 1. Introduction

Women are more susceptible to major depressive disorder (MDD) with a two-fold increased likelihood and a four-fold higher chance of having multiple depressive episodes compared to men (Kessler, 2003; Maciejewski et al., 2001; Seedat et al., 2009; Weissman et al., 1996). It has been reported that women has greater depression severity amongst other mood disorder-associated symptoms (Fava et al., 2003; Kornstein et al., 2000; Marcus et al., 2005; Shors et al., 2017). Despite the potential role of hormonal-related changes in depression having been investigated, the reasons behind sex/gender differences in depression remain poorly understood. This emphasizes the need for further research to understand the mechanisms associated to with sex/gender differences in MDD, especially since current antidepressant treatments are only effective for about 50 % of patients. It may be possible to develop gender specific treatments for MDD management.

Chronic stress has been identified as a significant factor to the onset and progression of MDD in both clinical settings and experimental mouse models (Breslau and Davis, 1986; Gururajan et al., 2019). Mouse models of chronic stress can provide valuable insight into the sex-specific neurobiological mechanisms underlying gender differences in MDD (Gururajan et al., 2019; Pesarico et al., 2021). Studies in preclinical and clinical settings have suggested that different brain regions, such as the medial prefrontal cortex (mPFC), are involved in sex-based differences in depression response to chronic stress (Belleau et al., 2019; Bittar and Labonte, 2021; Maeng and Shors, 2013). For instance, female rodents exposed to chronic stress have shown reductions in dendritic length and synapse number in the mPFC which is not seen in male rodents(Garrett and Wellman, 2009; Wohleb et al., 2018). Thus, sex/gender differences in response to chronic stress on synaptic plasticity and behavior may be influenced by microglia, since stress can alter their function.

Activated microglia play an important role in neuroinflammation, the cellular and biochemical responses to various insults (Schramm and Waisman, 2022; Woodburn et al., 2021). Furthermore, clinical studies demonstrated that microglial activation is involved in the development and progression of depression (Wang et al., 2022). Microglia are uniquely attuned to neuronal activity and can orient their processes toward signs of distress, aiding neurons via the release of soluble factors, modulation of neurotransmission, phagocytosis of dendritic elements, and induction of synaptic growth (Bollinger and Wohleb, 2019). Microglia proximity to neurons and ability to regulate neuronal shape and function, allows them to serve as critical mediators of sex-specific stress effects on behavior which may be critical in MDD pathogenesis (Gaspar et al., 2022; Tsyglakova et al., 2021).

Toll-like receptor 4 (TLR4), predominantly expressed by microglia, has emerged as a crucial player in the pathophysiology of diverse health conditions, exerting its influence through the modulation of innate immune responses (Rahimian et al., 2022). Accordingly, TLR4 activation has been implicated in neuroinflammatory responses and has shown associations with MDD (Gárate et al., 2019; Liu et al., 2019). Recent ´ studies have also highlighted the involvement of TLR4 in modulating synaptic plasticity, neurogenesis, and behavior in response to chronic stress (Hou et al., 2019; Wang et al., 2020). However, the specific role of TLR4 in the context of sex/gender differences in depressive-like behavior and stress remains poorly understood.

Given the higher risk for depression in women, we hypothesize that sex-dependent differences in depression susceptibility in response to chronic stress may be owing to the divergent lasting stress-induced microglia-related neuroinflammation in the mPFC. The present study was designed to explore how activated microglia influences persistent depression-like behavior in response to chronic stress in a sex-specific way.

## 2. Materials and methods

### 2.1. Animals

C57BL/6, Cx3Cr1^CreEr^, Eef1a1^LSLeGFP10a/+^, Tlr4^fl^ mice were purchased from The Jackson Laboratory. Eef1a1^LSLeGFP10a/+^ or Tlr4^fl^ mice and crossed with Cx3Cr1^CreEr^ mice. For genotyping of Cx3Cr1^CreE^; Tlr4^fl^ double transgenic mice, the primers were used (Cx3Cr1^CreEr^-mutant reverse; 5′-CGGTTATTCAACTTGCACCA-3′, Cx3Cr1^CreEr^-WT reverse; 5′-AGGATGTTGACTTCCGAGTTG-3′, Cx3Cr1^CreEr^-common forward; 5′-AAGACTCACGTG GACCTGCT-3′, Tlr4^fl^ reverse; 5′-TGATGGTGTGAGCAGGAGAG-3′ and Tlr4^fl^ forward; 5′-TGACCACCCATATTGCCTATAC-3′). All mice were single housed with a 12-h light/dark cycle (lights on from 07:00 to 19:00 h) at constant temperature (23 °C) with ad-libitum access to food and water. All protocols were approved by IACUC at Icahn School of Medicine at Mount Sinai and were performed in accordance with NIH guidelines.

### 2.2. Chronic stress exposure

**Fig. 1.**
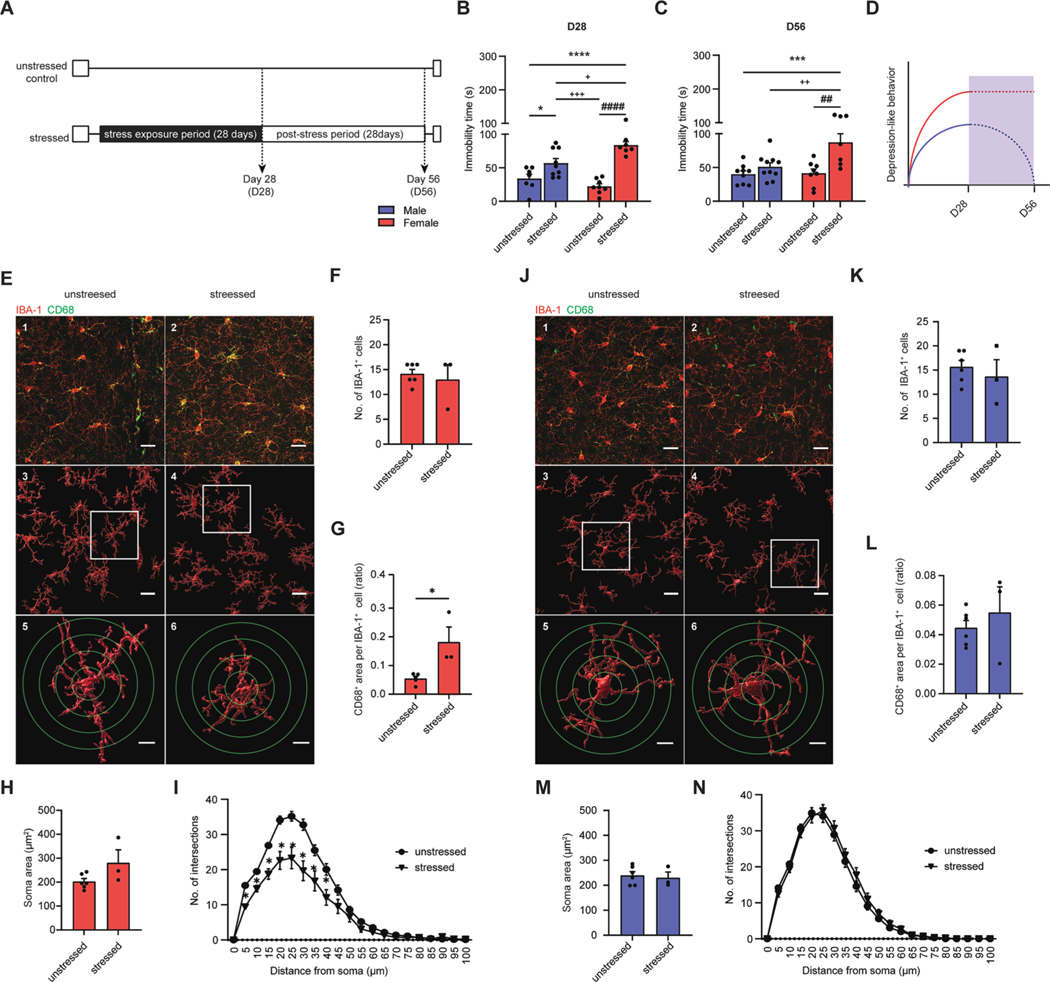
Female mice showed persistent depression-like behavior and changes in microglial morphology and increased activated phagocytic microglia. (A) Experimental outline. Unstressed mice that were not exposed to chronic stress and age-matched (upper panel) and stressed mice that were exposed to chronic stress exposure for 28 days, followed by 28 days of post-stress period. (B and C) Bar graphs showing total immobility time in female (red bar) and male (blue bar) stressed mice compared to unstressed mice at D28 (B) and at D56(C). Statistical analyses were performed using Two-way ANOVA (stress effect, *p* < 0.0001; sex effect, *p* = 0.2065; interaction effect, *p* = 0.0030; *p < 0.05, ****p < 0.001 compared to unstressed male mice; ^####^p < 0.0001, compared to unstressed female mice, ^+^*p* < 0.05, ^+++^*p* < 0.001, compared to stressed male mice, n = 7–9 mice for each group). (D) A graph showing depression-like behavior over the entire experimental, with female mice shown in red and male mice shown in blue. (E-N) Microglia activation and morphological analysis in female and male mice at D56. (E and J) Representative immunofluorescence images of IBA-1^+^ (red) and CD68^+^ (yellow) cells in female (E.1 and E.2) and male (J.1 and J.2) mice. (F and K) Bar graphs showing number of IBA-1^+^ cells in female (F) and male (K) mice. (G and L) Bar graphs showing quantification of CD68^+^area per IBA-1^+^ microglia in female (G) and male (L) mice. (E and J) Representative images of three-dimensional (3D) reconstruction in female (E.3, E.4, E.5 and E.6) male (J.3, J.4, J.5 and J.6) mice. The outlined with a white box is magnified. (H-N) Sholl analysis based on 3D reconstruction in female and male mice. Bar graph showing soma size per microglia in female (H) and male (M) mice. Line graphs showing quantification of number of intersection between each circle (green, E5, E6, J5 and J6) in female (I) and male (N) mice. Statistical analyses were performed using *t*-test (*p < 0.05, compared to unstressed female mice). Scale bars indicate 20 (E and J.1–2) and 10 (E and J.3–6) um. Each dot represents an individual mouse. Results are expressed as the mean ± SEM.

For assessing sex differences in a model of chronic stress, 2-month-old male and female mice including both C57BL/6 and Cx3Cr1^CreEr^; Tlr4^fl^ double transgenic mice were randomly subdivided for treatment groups including; non-stressed mice and stressed mice exposed to chronic stress (28-days, D28), followed by post-stress period (28-days, D56) as depicted in Fig. 1A and Fig. 3A. During the stress exposure periods, animals in all experimental groups, were subjected twice daily to random mild stressors (Table S2), as previously described (Westfall et al., 2021). Briefly, stressors included crowding with 12 animals/cage for 1 h, cage shaking for 20 min, 45° cage tilt for 10 h, 4 °C cold or 30 °C hot exposure for 1 h, food and/or water deprivation for 12 h, reversed light schedule, predator scent exposure for 10 h, cold water swim for 5 min, wet bedding or no bedding for 12 h, or social isolation for 1 h.

### 2.3. Tamoxifen treatment

2-month-old of Cx3Cr1^CreEr^; Tlr4^fl^ double transgenic mice were exposed to chronic stress for 28 days, followed by post-stress period and then, randomly divided into two groups, treated with either vehicle or tamoxifen to activate the tamoxifen-inducible mouse model. Tamoxifen powder (T5648, Sigma, MO, USA) was dissolved in corn oil (C8267, Sigma) with a concentration of 100 mg/mL. The prepared tamoxifen working solution was covered with aluminum foil and vigorously shaken at 37 °C for overnight and was then stored at 4 °C, protected from light. In accordance recent studies (Huck et al., 2021; Sahasrabuddhe and Ghosh, 2022), modified tamoxifen treatment regimen is proposed, aiming to enhance efficacy and minimize potential adverse effects. The proposed regimen involves administering mice with 6 doses of tamoxifen at 100 mg/kg, mixed with corn oil (C8267, Sigma), over a period of 12 days, with each dose separated by 48 h (Fig. 3A).

### 2.4. Behavior tests

#### 2.4.1. Forced swimming test

The forced swim test, a behavioral test in rodents, was employed to assess depression-like behaviors and evaluate the efficacy of antidepressant interventions (Yankelevitch-Yahav et al., 2015). All mice were allowed to accommodate to the behavioral room for 30 min before the starting of the behavioral testing. Male and female mice were tested in random order on the same day of experiment. Mice were individually put into 15-cm-diameter glass cylinder filled to 20 cm with 23 ± 1 °C water and cylinders were cleaned after every testing session. Immobility, defined as the absence of all movement except motions required maintaining the animal’s head above the water, was independently evaluated from video clips during a 6-min session. Results were expressed as time (in seconds) that animals spent immobile during the final 5-min. Animal behavior was video-recorded, tracked, and analyzed with ANY-maze^™^ tracking software (Steeling Co., IL, USA).

#### 2.4.2. Open field test

The Open field test involved placing mice into a 40 × 40×40 cm box in a brightly lit room with an intensity of 150 lx (Seibenhener and Wooten, 2015). The test lasted for 10 min, during which the mice’s total distance traveled were recorded. The mice’s behavior was video-recorded, and ANY-maze^™^ tracking software was used to track and analyze their movements.

### 2.5. Tissue preparation

After the completion of the last behavioral tests at D56 or D68, all mice from each experimental group underwent perfusion for brain collection. Randomly selected brain tissues were dissected into two regions: the prefrontal cortex (PFC) and the other hemisphere. These tissues were utilized for molecular experiments such as TRAP and western blot analysis. Additionally, brain tissue from the remaining mice was fixed with 4 % paraformaldehyde (PFA) for 24 h to facilitate immunofluorescence studies. All collected samples were appropriately stored at either −80 °C or 4 °C until further analysis.

### 2.6. TRAP and bioinformatics analysis

This approach relies on the genetic labeling of the ribosomal protein L10a with the enhanced green fluorescent protein (GFP) in a cell-type-specific fashion followed by GFP-based immune-affinity purification of the ribosome-associated mRNAs. To generate microglia-specific TRAP mice, mice that carry a lox-flanked STOP cassette (LSL) upstream of the eGFP-L10a gene under the control of eukaryotic translation elongation factor 1 alpha 1, Eef1a1 (Eef1a1^LSL eGFPL10a^), were bred to mice that express the Cre recombinase under the control of the microglia-/macrophage specific gene promoter, *Cx3Cr1*. Ribosome- associated mRNA from microglia was isolated from mPFC as previously described (Heiman et al., 2014). Briefly, all TRAP experiments were performed using dissected brain tissues that were frozen in liquid nitrogen. Brain tissue from one mouse was immediately homogenized with a motor-driven Teflon glass homogenizer in ice-cold polysome extraction (10 mM HEPES (pH 7.3), 150 mM KCl, 5 mM MgCl2, 0.5 mM dithiothreitol, 100 μ g/mL cycloheximide, EDTA-free protease inhibitor cocktail, 10 μ L/mL RNasin and Superasin. Homogenates were centrifuged for 10 min at 2,000g, 4 °C, to pellet large cell debris. NP-40 and 1, 2-diheptanoyl-*sn*-glycero-3-phosphocholine were added to the supernatant at final concentrations of 1 % and 30 mM, respectively. After incubation on ice for 5 min, the lysate was centrifuged for 10 min at 13,000g to pellet insoluble material. Goat anti GFP (19C8 and 19F7, Antibody & Bioresource Core Facility, NY, USA) and biotinylated Protein L (GenScript, NJ, USA)-coated Streptavid in MyOne T1 Dynabeads were added to the supernatant, and the mixture was incubated at 4 °C with end-over-end rotation overnight. Beads were collected on a magnetic rack and washed four times with high-salt polysome wash buffer (10 mM HEPES (pH 7.3), 350 mM KCl, 5 mM MgCl2, 1 % NP-40, 0.5 mM dithiothreitol, and 100 μ g/mL cycloheximide). RNA was purified from beads directly using RNeasy Mini Kit following the manufacturer’s instructions. RNA integrity was assayed using an RNA Pico chip on a Bioanalyzer 2100 and only samples with RIN > 9 were considered for subsequent analysis. TRAP and unbound fraction were sent to GENEWIZ to produce libraries for sequencing by NextSeq 500 (Illumina) with high-output single read sequencing for 75 cycles. Raw sequencing data was processed using Illumina bcl2fastq2 Conversion Software v2.17. Raw sequencing data was processed using the NGS-Data-Charmer pipeline, available in Github (https://github.com/shenlab-sinai/NGS-Data-Charmer). In brief, the sequenced reads were trimmed to remove adaptor and low-quality bases. The trimmed reads were then aligned to the mm10 mouse genome. Read counts within each gene were calculated with feature counts, using parameters (-p –O –fraction -t “gene”), and the comprehensive gene annotation from Gencode (vM25) for the murine genome (GRCm38). Samples with qualitatively poor TRAP enrichment of the microglia cell type were removed from downstream analyses and visualization. DESeq2 (v1.32.0) was used in R (v 4.1.1) to identify genes enriched in the TRAP condition, when compared to the UB condition, in either the FC brain regions. To identify genes differentially expressed between specific sample groups, TRAP-enriched genes were used as input for differential expression analysis with DESeq2. Ontological analysis was performed using the ‘enrichR’ package (v3.0), using the following four databases: “Mouse Gene Atlas”, “GO Molecular Function 2015”, “GO Cellular Component 2015”, and “GO Biological Process 2015”databases. Murine cell-type specific gene sets were obtained from a previous publication. A variance stabilizing transformation (VST) was applied to the count data, and all visualizations were generated using the VST-normalized values.

### 2.7. Immunofluorescence and sholl analysis

Fixed brains using 4 % paraformaldehyde (Electron Microscopy Sciences) were removed and dehydrated in PBS. Brain sections (5 μm) were washed with PBS, permeabilized with PBS + 0.2 % Triton X-100 (PBST) followed by blocking with 2 % normal goat serum in PBST. Brain sections were incubated with primary antibodies 2 % normal goat serum in PBST overnight at 4 °C. Primary antibodies: IBA1 (1:1000, 019–19741, Wako Chemicals, VA, USA), CD68 (1:1000, MCA1957, Bio-Rad, CA, USA). Brain sections were then washed and incubated with Alexa Fluor-conjugated secondary antibodies at 1:500 in 2 % normal goat serum in PBST for 1 h at RT. Secondary antibodies are Alexa Fluor 488-labeled and Alexa Fluor 568-labeled antibodies. Brain sections were washed and mounted and coverslipped on microscope slides using Vecta Shield containing DAPI (H-1500, Vector Laboratories, CA, USA). Imaging was performed using a Zeiss LSM 880 Confocal Microscope (Zeiss, DE, Germany). The counts of microglia were analyzed using ImageJ software (National Institutes of Health). The co-localization and quantification of IBA1 or CD68 immunopositve cells protein in brain sections from the mPFC of male and female mice were determined using IMARIS 9.1.2 (Bit Plane Inc, MA, USA).The following steps were taken for image processing: surface images were created separately for IBA1^+^ microglia cells and CD68^+^ protein. The IMARIS 9.5 Global filtering native distance measure was then used to determine the co-localization of CD68 surfaces, using a random color mask for each IBA-1^+^ microglia cell (12 to 20 cells per sample). To quantify the expression of CD68^+^ in microglia, the surface-surface overlap percentage was calculated, and the averages were determined among samples within the same group. For 3D analysis, z-stack confocal images were processed using AutoQuant X3.1 (Media Cybernetics, MD, USA) to remove any blurriness and then analyzed using IMARIS. The surface tool was used to reconstruct the cell bodies (somas) of the microglia, while the filaments tool was used to reconstruct the branches. One microglial cell was selected per total process area, and the cell body and soma were traced along with the surface and filament for each process. The number of primary processes and branch tips were counted. To determine the complexity of each cell, a sholl analysis was performed. One microglial cell was selected per 0.045 μm^2^ of each images. Concentric circles were drawn with 5 μm spacing originating from the soma, and the number of intersections of each cell with each circle was measured. The analysis was performed on 3 to 6 mice (15–20 microglia cells per mouse) per group. The immunocytochemical experiments were carried out by a single investigator, while an independent evaluator performed the quantification analysis in a blinded manner, utilizing code that prevented any knowledge of the experimental groups. Microscopy and image analysis was performed at the Microscopy CoRE at the Icahn School of Medicine at Mount Sinai.

### 2.8. Western blotting

To analyze protein samples from the PFC, the samples were extracted and then separated by electrophoresis on a 4 % to 15 % sodium dodecyl sulfate–polyacrylamide gel. The separated proteins were then transferred to a nitrocellulose membrane. The membrane was then blocked for 1 h at room temperature and treated overnight with primary antibodies specific to p-NF-kB p65 (3033 s, Abcam), NF-kB p65 (51–0500, Thermo Fisher Scientific), caspase-1 (20B-0042, adipogen, CA, USA), IL-1β (6243S, Cell signaling, MA, USA), and α-tubulin (T9026, Sigma) at 4 °C. The following day, the blots were exposed to a secondary antibody conjugated with horseradish peroxidase (anti-rabbit-IgG-antibody; 31460, Thermo Fisher Scientific or anti-mouse IgG-antibody; ab6808, Abcam) for 1 h at room temperature. The resulting bands were detected using a chemiluminescence detection kit (32106; Thermo Fisher Scientific, MA, USA) and the data was analyzed using ImageJ software to determine the relative expression of each protein.

### 2.9. Enzyme-linked immunosorbent assay (ELISA)

The measurement of IL-1β in the PFC was performed using the Mouse IL-1β/IL-1F2 DuoSet ELISA Kit (DY201–05, R&D Systems) in accordance with the manufacturer’s instructions. The absorbance at 450 nm was then recorded using a microplate reader (Varioskan LUX, Thermo Fisher Scientific).

### 2.10. Statistical analysis

All values are expressed as mean and standard error of the mean (S.E. M). Data were calculated using a *t*-test and One or Two-way analysis of variance (ANOVA) followed by Tukey’s post hoc analysis (*p < 0.05, **p < 0.01, ***p < 0.001). All statistical analysis was performed using GraphPadPrism 5 software (Graph Pad Software, Inc.).

## 3. Results

### 3.1. Differential sex-associated behavior response to chronic stress

To investigate the effects of chronic stress on sex associated depression-like behavior, male and female mice were subjected to 28-days of chronic stress, followed by post-stress period of 28-days (Fig. 1A) based on a previously optimized protocol (Sequeira-Cordero et al., 2019; Westfall et al., 2021). Depression-like behavior was evaluated by measuring immobility time, which is often considered to reflect a measure of behavioral despair, using the forced swimming test (FST) (Cryan and Holmes, 2005), at both D28 (Fig. 1B) and D56 (Fig. 1C). We find that immobility time was significantly increased in both male and female-stressed mice compared to unstressed control mice at D28 (Fig. 1B, *p < 0.05, ^####^*p* < 0.0001). Moreover, female-stressed mice showed a significantly higher increase in immobility time compared to male-stressed mice at D28 (Fig. 1B, ^+++^*p* < 0.001). Interestingly, only female stressed mice showed a significant 1.3-fold increase in immobility time at D56 compared to unstressed control mice (Fig. 1C, ^##^*p* < 0.01), while male mice reached to bassline level at D56 (Fig. 1C). In addition, neither male nor female mice exposed to chronic stress showed any significant changes in locomotor activity compared to unstressed control mice at D28 (Supplementary Fig. 1A) or D56 (Supplementary Fig. 1B). The results indicate that chronic stress leads to a stronger depression-like behavior in female mice compared to male mice, and the effects of chronic stress on depression-like behavior persisted in female mice even after post-stress period (Fig. 1D).

### 3.2. Differential sex-associated microglia activation which is related to persistent depression-like behavior

The mPFC, a central region in emotional processing and response to stress, have been implicated in MDD and stress-induced depression-like behavior (Bittar and Labonte, 2021). To investigate microglia activation in response to chronic stress followed by post-stress period mPFC in both female and male mice, we first measured the number of IBA-1^+^ microglia cells (Fig. 1F and 1 K). We find no significant change in the number of IBA-1^+^ microglia cells in mPFC of both female (Fig. 1F) and male (Fig. 1K)-stressed mice compared to unstressed control mice. Then, we further examined whether activated phagocytic microglia are altered at D56 in both female and male mice. Using double immunostaining of CD68 and IBA-1, which allowed us to distinguish between resting and activated phagocytic microglia (Hopperton et al., 2018), we assessed the activated phagocytic microglia in the mPFC (Fig. 1E and 1 J). We find that the ratio of CD68^+^ area per IBA-1^+^ microglia cells in the mPFC was significantly higher in female-stressed mice compared to unstressed female mice at D56 (Fig. 1G, *p < 0.05). The ratio of CD68^+^ area per IBA-1^+^ microglia cells in the mPFC did not differ significantly between stressed and unstressed male mice (Fig. 1L). Collectively, these results suggest that the microglia activation in female in mPFC, but not in the male, persisted at D56, which coincides with the depression-like behavior.

To explore sex differences in the microglial morphological phenotypes, such as enlargement of soma size and length of branches from the soma in the PFC at D56, a sholl analysis was performed using 3-dimensional(3D) morphometric analysis with IMARIS software (Fig. 1E and 1 J). The soma size of IBA-1 immunopositive microglia did not differ significantly in female-stressed mice compared to unstressed control mice at D56 (Fig. 1H). Interestingly, the analysis revealed a significant reduction in branch length from the soma of IBA-1 immunopositive microglia in female-stressed mice compared to unstressed control mice (Fig. 1I, *p < 0.05). However, the soma size (Fig. 1M) and the branch length (Fig. 1N) of microglia did not exhibit significant differences in male mice exposed to chronic stress compared to unstressed control mice. This evidence suggests tentatively that in females, the complexity of microglia processes was decreased, which was coincided with persistent depression-like behavior at D56.

### 3.3. Differential sex-associated microglia-specific gene expression

**Fig. 2.**
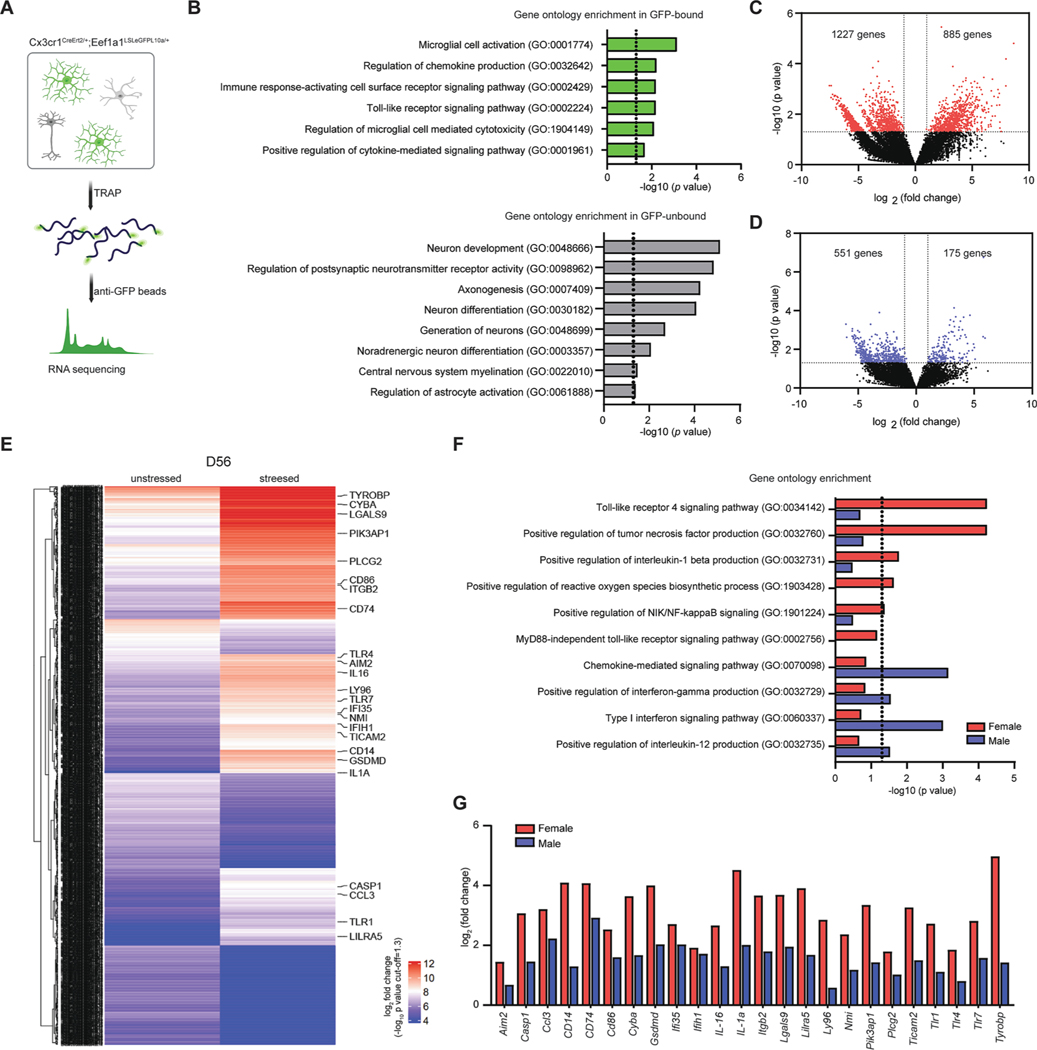
Female mice showed alteration of the expression of microglia-specific mRNAs linked to the TLR4 signaling pathway. (A) Schematic showing TRAP-sequencing. (B) Selected Gene ontology (GO) annotations enriched in GFP-bound (green, upper panel) and GFP-unbound group (gray, lower panel).The x-axis represents - log10 (p-value), with dotted line representing a *p*-value of 0.05. (C-D) Volcano plots in female (C) and male (D) mice. The x-axis represents the log_2_ conversion of the fold change (log_2_ FC) values, and the y-axis represents the corrected significance level after base log10 conversion (*p* value). Red and blue dots in the volcano plot indicate all DEGs that were found to differ significantly (absolute value of log_2_ FC > 1, *p < 0.05). (E) Heat map with hierarchical clustering distances shows the variation in the expression levels (VST-scored log_2_ RPKM) between female stressed and unstressed mice. (F) A bar graph showing selected GO annotations enriched in female (red) and male (blue) mice. The x-axis represents -log10 (*p*-value), with dotted line representing a *p*-value of 0.05. (G) A bar graph showing log_2_ FC of significant DEGs in female stress (red) and male stress (blue) mice using a cut-off of *p* < 0.05.

To explore the specific molecular mechanisms in microglia underlying persistent depression-like behavior at D56, following chronic stress, translating-ribosome affinity purification (TRAP) (Heiman et al., 2014) was used by utilizing double transgenic mice including Cx3Cr1^CreErt2^ (Cx3Cr1^CreEr^) and Eef1a1^LSLeGFP10a/+^ strain (Fig. 2A). To confirm the specificity of TRAP method in isolating microglial RNA, we analyzed gene ontology (GO) from the GFP-bound and GFP-unbound RNA. By comparing the enriched GO categories in the GFP-bound and GFP-unbound, we find that the TRAP method effectively isolated microglial RNA, as indicated by the enrichment of microglia-associated GO in the GFP-bound (Fig. 2B, upper panel) and the enrichment of neuron, astrocyte or oligodendrocyte-associated GO in the unbound (Fig. 2B, bottom panel). Based on this, we proceeded to identify differentially expression genes (DEGs) changes in microglia of the mPFC in female and male-stressed mice compared to unstressed control mice at D56.

A total of 16,595 DEGs in female and male stressed mice were identified compared to unstressed mice and displayed as a volcano plot (Fig. 2C and 2D). Of these 16,595 DEGs, 885 DEGs were found to be up-regulated and 1227 DEGs were down-regulated significantly with an absolute value of log_2_ fold change (FC) greater than 1 and a p-value less than 0.05, as shown in Fig. 2C. In male-stressed mice, only 175 DEGs were found to be up-regulated and 551 DEGs were down-regulated significantly (absolute value of log_2_ FC > 1, p < 0.05, Fig. 2D). These findings suggest that female-stressed mice showed more significant changes in gene expression compared to male-stressed mice at D56. Additionally, the heatmap of whole-transcriptome gene expression presents clear visual evidence of a separation of molecular signatures between stressed and unstressed female mice (Fig. 2E).

Subsequently, functional enrichment analysis was conducted on the significant DEGs in both the female and male groups to understand the underlying biological processes (Fig. 2F and 2G). As shown Fig. 2F, GO analysis showed that the common TOP 10 of GO were observed in both female and male-stressed mice at D56. Notably, microglia the mPFC of female stressed mice showed 3 significant enrichments related with stress-inducing immunological priming signaling including ‘*Toll-like receptor 4 signaling pathway, Positive regulation of NIK/NF-kappa signaling, and MyD88-independent toll-like receptor signaling pathway*’. Additionally, there was enrichment in signaling associated with the formation and secretion of IL-1β such as ‘*Positive regulation of interleukin-1 beta production and Positive regulation of tumor necrosis factor production*” (red bar, Fig. 2F, p < 0.05). In contrast, microglia the mPFC of male stressed mice were enriched associated with moderate microglia-specific priming, including *Type I interferon signaling pathway and Positive regulation of interferon-gamma production’* (blue bar, Fig. 2F, p < 0.05). We also find that the expression of 24 DEGs in the top 5 canonical pathways, which were significantly enriched in only female-stressed mice, were also elevated in males-stressed mice, but to a lesser extent (Fig. 2G, *p* < 0.05). This evidence indicates that there might be a sex difference in the molecular mechanisms of microglia activation in response to chronic stress. This possibility is suggested by the fact that female mice showed a greater abundance of microglia-specific genes, particularly those linked to the TLR4 signaling pathway, in microglia in the mPFC.

### 3.4. The mitigation of persistent depression-like behavior caused by the genetic ablation of TLR4 expression in microglia after post-stress period

**Fig. 3.**
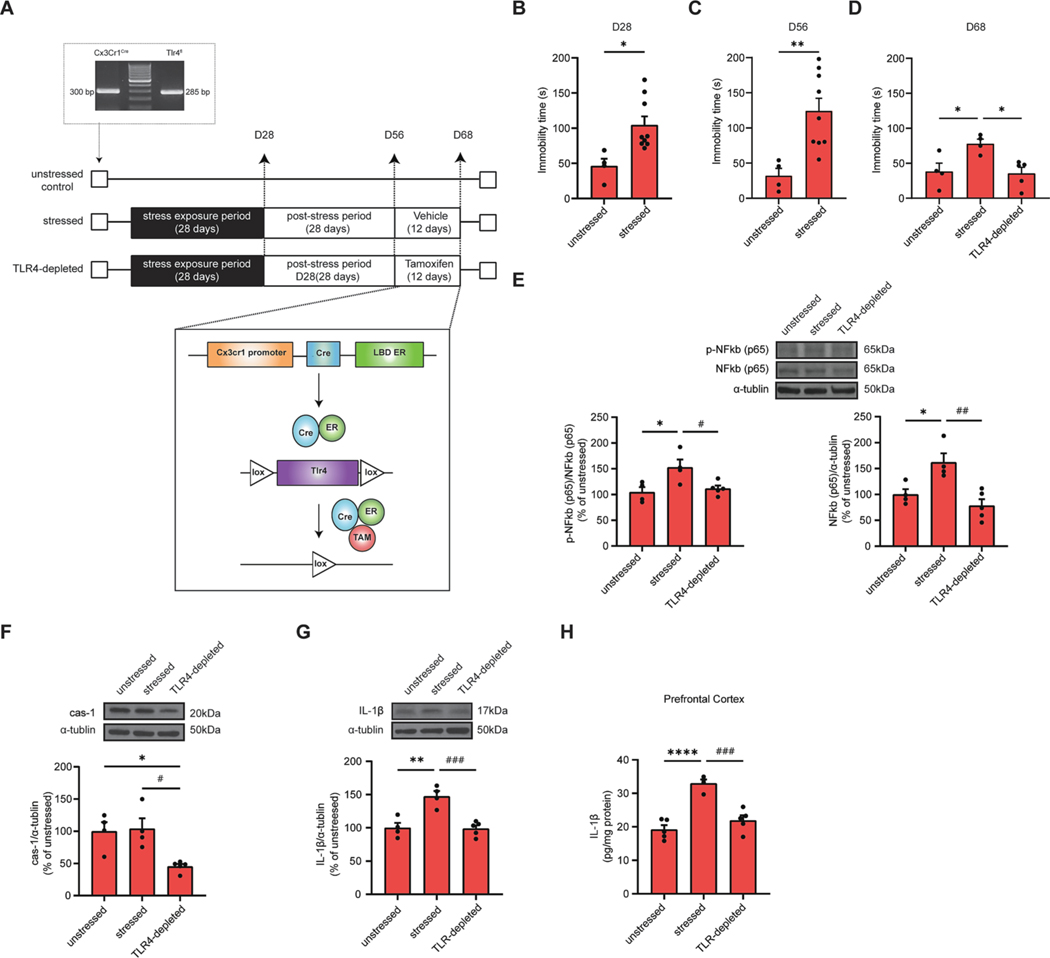
Female mice with blocked TLR4 signaling pathway showed lessened persistent depression-like behavior following chronic stress. (A) The experimental outline in Cx3Cr1^CreEr^; Tlr4^fl^ double transgenic mice. Unstressed female mice that were not exposed to chronic stress and age-matched and stressed female mice that were exposed to chronic stress for 28 days, followed by 28 days of post-stress period, and then treated with vehicle or tamoxifen for an additional 12 days. (B-D) Bar graphs showing total immobility time at D28 (B), D56 (C) and D68 (D). (E-G) Representative western blot of p-NF-KB 65 (E, upper panel), NF-kB p65 (E, upper panel), caspase-1 (F, upper panel) and IL-1β (G, upper panel) and bar graphs showing relative quantification of p-NF-KB p65 (E, bottom left panel) normalized to NF-kB p65, NF-kB p65 (E, bottom right panel), cleaved caspase-1 (F, bottom panel) and IL-1β (G, bottom panel) levels normalized to α-tubulin. (H) Bar graph showing concentration of IL-1β in PFC lysates. Statistical analyses were performed using *t*-test or One-way ANOVA (*p < 0.05, **p < 0.01, ****p < 0.0001 compared to female unstressed mice; ^#^*p* < 0.05, ^##^*p* < 0.01, ^###^*p* < 0.001, compared to female stressed mice, n = 4–5 mice for each group). Each dot represents an individual mouse. Results are expressed as the mean ± SEM.

To investigate the role of TLR4 signaling pathway on microglia in female mice with persistent depression-like behavior, we generated a double transgenic mouse by crossbreeding Cx3Cr1^CreEr^ and Tlr4^tm1/1Karp^ (Tlr4^fl^) mice. Female mice were exposed to 28-days of chronic stress, followed by a 28-days of post stress period and then treated with tamoxifen for 12 days to suppress TLR4 signaling pathway in microglia (Fig. 3A). As expected, we find that the immobility time was significantly increased in female-stressed mice compared to unstressed control mice at D28 (Fig. 3B, *p < 0.05) and D56 (Fig. 3C, **p < 0.01). Interestingly, persistent depression-like behavior was significantly mitigated, strongly implicating the causal role of TLR4 signaling pathway in microglia of female mice in response to chronic stress (Fig. 3D, *p < 0.05). Additionally, we observed that male mice exhibited depression-like behavior after stress exposure at D28 (Supplementary Fig. 2A, **p < 0.01). However, this depression-like behavior was not observed in male mice at D56 (Supplementary Fig. 2B). Consequently, no noticeable differences were observed between stressed mice and TLR4-depleted mice within the male group at D68 (Supplementary Fig. 2C).

In order to evaluate innate immune response in PFC of female mice subjected to chronic stress via TLR4-Nuclear factor kappa-light-chain-enhancer of activated B (NF-kB) signaling, the protein expression levels of NF-kB was measured. As shown in Fig. 3E, stressed female mice exhibited significant elevation in protein level of phosphorylation of NF-kB p65 compared to unstressed mice (*p < 0.05). Notably, the enhanced phosphorylation of NF-kB p65 protein expression was significantly mitigated by ablation of TLR4 (Fig. 3E, left panel, ^#^*p* < 0.05). Interestingly, this change coincided with a significant alteration in the protein expression of NF-kB p65, suggesting that stress activated the NF-kB signaling, which was subsequently inhibited by TLR4 ablation in female mice exposed to stress. (Fig. 3E, right panel, *p <0.05, ^##^*p* <0.01). Additionally, we analyzed the protein expression of Caspase-1 and IL-1β, as these are downstream of inflammasome activation triggered by TLR4-NF-kB signaling (Fig. 3F and 3G). As shown in Fig. 3F, the expression of cleaved caspase-1 was not higher in female-stressed mice compared to unstressed mice. However, a reduction in cleaved caspase-1 levels was observed in TLR4-depleted mice compared to female-stress mice and unstressed mice (Fig. 3F, *p < 0.05, ^#^*p* < 0.05). Similarly, through western blot analysis, TLR4-depleted female mice exhibited a significant inhibition of protein expression of IL-1β, one of the major players in the downstream of inflammasome activation in the PFC compared to female-stressed mice (Fig. 3G, **p < 0.01, ^###^*p* < 0.001). Moreover, these findings were substantiated by ELISA, which consistently revealed a reduction in the levels of secreted IL-1β in TLR4-depleted female mice subjected to stress (Fig. 3H, ****p < 0.0001, ^###^*p* < 0.001). Consistent to This evidence tentatively suggests that TLR4 signaling in microglia may have an important role in persistent depression-like behavior in female mice.

## 4. Discussion

Women are twice as likely as men to develop serious stress-related disorders, such as MDD, and are more likely to suffer from recurrent depression due to the impact of stress experienced over lifetimes (Burcusa and Iacono, 2007; Essau et al., 2010; Goodwill et al., 2019; Oquendo et al., 2013). Although depression is more common among women, only a limited number of studies have examined the issue of gender differences and the response to psychopharmacological treatment in MDD, particularly in those who have treatment-resistant depression (Bartova et al., 2021; LeGates et al., 2019; Moderie et al., 2022; Rubinow and Schmidt, 2019). In the present study, we find that chronic stress induces depression-like behavior in both male and female mice, which was evaluated using the FST, also known as a behavioral despair test (Cryan and Holmes, 2005). Notably, female mice exhibited longer depressive-like behavior up to one month after chronic stress period, by which time male mice had already ceased displaying depression-like behavior toward unstressed control mice (Fig. 1). Our results provide evidence that exposure to chronic stress in female mice can trigger alternative mechanisms that may contribute to persistent depression-like behavior. This is consistent with previous studies that have shown a higher incidence of MDD and refractory responses to certain antidepressants in women (Bartova et al., 2021; LeGates et al., 2019; Maciejewski et al., 2001). The consistency between our study and prior research highlights the importance of adopting a sex-specific approach in understanding and addressing MDD. These findings underscore the need for tailored interventions that recognize and account for the unique experiences and characteristics of individuals based on their sex or gender in the diagnosis and treatment of MDD.

Previous studies have suggested a link between inflammation in microglia and MDD (Miller and Raison, 2016). Based on this, we hypothesize that the differential cellular and molecular responses in activated microglia may contribute to persistent depression-like behavior in a sex-dependent manner. Our study provides evidence of a persistent activation of microglia that is associated with sex, characterized with short branching and ramified appearance in the mPFC. This persistent phenotype during post-stress period, coincided with the persistent depression-like behavior in female mice. In contrast, microglia activation was not observed in male mice (Fig. 1 and Fig. 2).

Recent studies have shown that chronic stress is associated with microglia-mediated neuronal remodeling and modulation of synaptic transmission which have been associated with microglia activation (Bollinger, 2021; Bollinger and Wohleb, 2019; Gerhard et al., 2016; Perry et al., 2010; Wohleb et al., 2016; Wohleb et al., 2018). In addition, the neurons within the mPFC, with its complex laminar and anatomical distribution, is ideally organized to integrate the diverse sensory and emotional information from other brain regions and redirecting it to other structures (including hippocampus, lateral habenula, and nucleus accumbens) to drive behavioral responses to stress (Bittar and Labonte, 2021). Our evidence of increased CD68 immunopositive microglia in mPFC, a marker of activated phagocytic microglia, at D56 in female is consistent with this hypothesis (Fig. 1). Our evidence tentatively suggests that the protracted microglia activation at D56 in a sex dependent manner could explain the lack of response to current available therapies aimed at reversing circuit abnormalities in depression which are affected by alternative mechanisms.

Based on our evidence showing activated phagocytic microglia in female mice with persistent depression-like behavior, we aim to further clarify the molecular mechanisms. Towards this goal, we utilized the TRAP technique to identify specific molecular mechanisms in microglia (Doyle et al., 2008; Heiman et al., 2008). This approach enables us to avoid artifacts associated with procedures that require the isolation of microglial cells (Doyle et al., 2008). Our findings revealed during post-stress period, female mice were characterized a significant activation of microglial TLR4 signaling pathway (Fig. 2). To investigate the causal effect of the TLR4 signaling pathway in microglia in persistent depression-like behavior in female mice, we genetically ablated TLR4 expression in a double transgenic mouse model. Interestingly, the persistent depression-like behavior was significantly reduced, strongly implicating the causal role of the TLR4 signaling pathway in microglia in the stress response of female mice (Fig. 3). Previous research has demonstrated that after exposure to severe short-term stressors (lasting less than 24 h), there is an increase in murine TLR4 expression in the hippocampal formation (Cheng et al., 2016). This evidence, along with our present results, strongly support the hypothesis that the activation of TLR4 signaling in microglia may play a causal role in stress-induced priming and persistence of depressive-like behaviors in a sex-dependent manner, possibly depending on the duration and frequency of stressors experienced.

Regulating neuroinflammation via pharmacological or genetic manipulation of the TLR4 signaling pathway can alter the impact of stress on behavior (Liu et al., 2014). TLR4 is a member of the Toll-like receptor family and is a part of the pattern recognition receptor family (Armant and Fenton, 2002). Activation of TLR4 triggers an intracellular signaling pathway involving NF-κB, which leads to the production of inflammatory cytokines and activates the innate immune system, including the inflammasome activation (O’Neill et al., 2013). Inflammasome activation leads to the activation of caspase-1, which in turn results in the production and release of IL-1β, a potent pro-inflammatory cytokine (Walsh et al., 2014). To determine whether inflammasome activation in response to chronic stress can be mitigated by ablation of TLR4 expression in female-stressed mice, we analyzed IL-1β expression in the mPFC at D68. We find a significant decrease in IL-1β expression in TLR4-depleted female mice, indicating that TLR4-mediated innate immunity in microglia may play a role in the persistent depression-like behavior in a sexually dependent manner.

The present study has several limitations that warrant acknowledgment and discussion. Firstly, our focus was to understand the connection between stress, microglia activation, and behavior in a sex-specific manner. We report causal effect relationship of TLR4 and microglia activation in sex-dependent manner associated with persistent depression-like behavior in females. Further research should also incorporate correlation and morphometric analysis to explore the potential associations between TLR4-related pathways, such as MyD88, NF-kB, and inflammasome signaling downstream pathways, in order to elucidate the underlying mechanisms of sex differences in proinflammatory changes within microglia. Additionally, the hormonal response to stress via the hypothalamic–pituitary–adrenal axis should also be explored in relation to persistent proinflammatory conditions after post-stress period and other stress-related outcomes in MDD, such as anxiety. Finally, it is important to acknowledge that the TLR4 deletion in this study had broader effects on brain regions implicated in depression, including the hippocampus, amygdala, nucleus accumbens, and others, rather than being specific to the prefrontal cortex (PFC). While our findings provide valuable insights into the role of TLR4 in sex-dependent behavioral phenotypes, further research is necessary to gain a better understanding of the specific neural circuits within the PFC that are involved in these behaviors following TLR4 deletion. Investigating the role of inflammation in these brain regions could help shed light on the underlying sex-dependent mechanisms involved during the post-stress period following the onset of depression. These findings have the potential to illuminate the underlying sex-dependent mechanisms involved in the post-stress period, shedding light on the processes following the onset of depression.

In conclusion, our study tentatively provides new evidence that could lead to new clinical interventions in a sex dependent manner to alleviate persistent depression in female through modulation of innate immunity in microglia.

## Supplementary Material

Supple 1

Supple 2

Supple 3

## Data Availability

Data will be made available on request.

## References

[R1] Armant MA, Fenton MJ, 2002. Toll-like receptors: a family of pattern-recognition receptors in mammals. Genome Biol 3, REVIEWS3011. 10.1186/gb-2002-3-8-reviews3011 PMC139401 12186654

[R2] Bartova L, Dold M, Fugger G, Kautzky A, Mitschek MMM, Weidenauer A, Hienert MG, Frey R, Mandelli L, Zohar J, Mendlewicz J, Souery D, Montgomery S, Fabbri C, Serretti A, Kasper S, 2021. Sex-related effects in major depressive disorder: Results of the European Group for the Study of Resistant Depression. Depress. Anxiety 38, 896–906. 34110066 10.1002/da.23165 PMC8453858

[R3] Belleau EL, Treadway MT, Pizzagalli DA, 2019. The Impact of Stress and Major Depressive Disorder on Hippocampal and Medial Prefrontal Cortex Morphology. Biol. Psychiatry 85, 443–453. 30470559 10.1016/j.biopsych.2018.09.031 PMC6380948

[R4] Bittar TP, Labonte B, 2021. Functional Contribution of the Medial Prefrontal Circuitry in Major Depressive Disorder and Stress-Induced Depressive-Like Behaviors. Front. Behav. Neurosci. 15, 699592. 34234655 10.3389/fnbeh.2021.699592 PMC8257081

[R5] Bollinger JL, 2021. Uncovering microglial pathways driving sex-specific neurobiological effects in stress and depression. Brain Behav Immun Health 16, 100320. 34589809 10.1016/j.bbih.2021.100320 PMC8474553

[R6] Bollinger JL, Wohleb ES, 2019. The formative role of microglia in stress-induced synaptic deficits and associated behavioral consequences. Neurosci. Lett 711, 134369. 31422099 10.1016/j.neulet.2019.134369 PMC9875737

[R7] Breslau N, Davis GC, 1986. Chronic stress and major depression. Arch. Gen. Psychiatry 43, 309–314. 2937384 10.1001/archpsyc.1986.01800040015003

[R8] Burcusa SL, Iacono WG, 2007. Risk for recurrence in depression. Clin. Psychol. Rev 27, 959–985. 17448579 10.1016/j.cpr.2007.02.005 PMC2169519

[R9] Cheng Y, Pardo M, Armini RS, Martinez A, Mouhsine H, Zagury JF, Jope RS, Beurel E, 2016. Stress-induced neuroinflammation is mediated by GSK3-dependent TLR4 signaling that promotes susceptibility to depression-like behavior. Brain Behav. Immun. 53, 207–222. 26772151 10.1016/j.bbi.2015.12.012 PMC4783243

[R10] Cryan JF, Holmes A, 2005. The ascent of mouse: advances in modelling human depression and anxiety. Nat. Rev. Drug Discov 4, 775–790. 16138108 10.1038/nrd1825

[R11] Doyle JP, Dougherty JD, Heiman M, Schmidt EF, Stevens TR, Ma G, Bupp S, Shrestha P, Shah RD, Doughty ML, Gong S, Greengard P, Heintz N, 2008. Application of a translational profiling approach for the comparative analysis of CNS cell types. Cell 135, 749–762. 19013282 10.1016/j.cell.2008.10.029 PMC2763427

[R12] Essau CA, Lewinsohn PM, Seeley JR, Sasagawa S, 2010. Gender differences in the developmental course of depression. J. Affect. Disord 127, 185–190. 20573404 10.1016/j.jad.2010.05.016 PMC3754427

[R13] Fava M, Rush AJ, Trivedi MH, Nierenberg AA, Thase ME, Sackeim HA, Quitkin FM, Wisniewski S, Lavori PW, Rosenbaum JF, Kupfer DJ, 2003. Background and rationale for the sequenced treatment alternatives to relieve depression (STAR*D) study. Psychiatr. Clin. North Am. 26 (457–494), x. 12778843 10.1016/s0193-953x(02)00107-7

[R14] Garrett JE, Wellman CL, 2009. Chronic stress effects on dendritic morphology in medial prefrontal cortex: sex differences and estrogen dependence. Neuroscience 162, 195–207. 19401219 10.1016/j.neuroscience.2009.04.057 PMC2720075

[R15] Gaspar R, Soares-Cunha C, Domingues AV, Coimbra B, Baptista FI, Pinto L, Ambrosio AF, Rodrigues AJ, Gomes CA, 2022. The Duration of Stress Determines Sex Specificities in the Vulnerability to Depression and in the Morphologic Remodeling of Neurons and Microglia. Front. Behav. Neurosci 16, 834821. 35330844 10.3389/fnbeh.2022.834821 PMC8940280

[R16] Gerhard DM, Wohleb ES, Duman RS, 2016. Emerging treatment mechanisms for depression: focus on glutamate and synaptic plasticity. Drug Discov. Today 21, 454–464. 26854424 10.1016/j.drudis.2016.01.016 PMC4803609

[R17] Goodwill HL, Manzano-Nieves G, Gallo M, Lee HI, Oyerinde E, Serre T, Bath KG, 2019. Early life stress leads to sex differences in development of depressive-like outcomes in a mouse model. Neuropsychopharmacology 44, 711–720. 30188513 10.1038/s41386-018-0195-5 PMC6372611

[R18] Gururajan A, Reif A, Cryan JF, Slattery DA, 2019. The future of rodent models in depression research. Nat. Rev. Neurosci 20, 686–701. 31578460 10.1038/s41583-019-0221-6

[R19] Heiman M, Schaefer A, Gong S, Peterson JD, Day M, Ramsey KE, Suarez-Farinas M, Schwarz C, Stephan DA, Surmeier DJ, Greengard P, Heintz N, 2008. A translational profiling approach for the molecular characterization of CNS cell types. Cell 135, 738–748. 19013281 10.1016/j.cell.2008.10.028 PMC2696821

[R20] Heiman M, Kulicke R, Fenster RJ, Greengard P, Heintz N, 2014. Cell type-specific mRNA purification by translating ribosome affinity purification (TRAP). Nat. Protoc 9, 1282–1291. 24810037 10.1038/nprot.2014.085 PMC4102313

[R21] Hopperton KE, Mohammad D, Trepanier MO, Giuliano V, Bazinet RP, 2018. Markers of microglia in post-mortem brain samples from patients with Alzheimer’s disease: a systematic review. Mol. Psychiatry 23, 177–198. 29230021 10.1038/mp.2017.246 PMC5794890

[R22] Huck NA, Siliezar-Doyle J, Haight ES, Ishida R, Forman TE, Wu S, Shen H, Takemura Y, Clark JD, Tawfik VL, 2021. Temporal Contribution of Myeloid-Lineage TLR4 to the Transition to Chronic Pain: A Focus on Sex Differences. J. Neurosci 41, 4349–4365. 33846230 10.1523/JNEUROSCI.1940-20.2021 PMC8143203

[R23] Kessler RC, 2003. Epidemiology of women and depression. J. Affect. Disord 74, 5–13. 12646294 10.1016/s0165-0327(02)00426-3

[R24] Kornstein SG, Schatzberg AF, Thase ME, Yonkers KA, McCullough JP, Keitner GI, Gelenberg AJ, Ryan CE, Hess AL, Harrison W, Davis SM, Keller MB, 2000. Gender differences in chronic major and double depression. J. Affect. Disord 60, 1–11. 10940442 10.1016/s0165-0327(99)00158-5

[R25] LeGates TA, Kvarta MD, Thompson SM, 2019. Sex differences in antidepressant efficacy. Neuropsychopharmacology 44, 140–154. 30082889 10.1038/s41386-018-0156-z PMC6235879

[R26] Liu J, Buisman-Pijlman F, Hutchinson MR, 2014. Toll-like receptor 4: innate immune regulator of neuroimmune and neuroendocrine interactions in stress and major depressive disorder. Front. Neurosci 8, 309. 25324715 10.3389/fnins.2014.00309 PMC4179746

[R27] Maciejewski PK, Prigerson HG, Mazure CM, 2001. Sex differences in event-related risk for major depression. Psychol. Med 31, 593–604. 11352362 10.1017/s0033291701003877

[R28] Maeng LY, Shors TJ, 2013. The stressed female brain: neuronal activity in the prelimbic but not infralimbic region of the medial prefrontal cortex suppresses learning after acute stress. Front. Neural Circuits 7, 198. 24391548 10.3389/fncir.2013.00198 PMC3868707

[R29] Marcus SM, Young EA, Kerber KB, Kornstein S, Farabaugh AH, Mitchell J, Wisniewski SR, Balasubramani GK, Trivedi MH, Rush AJ, 2005. Gender differences in depression: findings from the STAR*D study. J. Affect. Disord 87, 141–150. 15982748 10.1016/j.jad.2004.09.008

[R30] Miller AH, Raison CL, 2016. The role of inflammation in depression: from evolutionary imperative to modern treatment target. Nat. Rev. Immunol 16, 22–34. 26711676 10.1038/nri.2015.5 PMC5542678

[R31] Moderie C, Nunez N, Fielding A, Comai S, Gobbi G, 2022. Sex Differences in Responses to Antidepressant Augmentations in Treatment-Resistant Depression. Int. J. Neuropsychopharmacol 25, 479–488. 35167671 10.1093/ijnp/pyac017 PMC9211005

[R32] O’Neill LA, Golenbock D, Bowie AG, 2013. The history of Toll-like receptors - redefining innate immunity. Nat. Rev. Immunol 13, 453–460. 23681101 10.1038/nri3446

[R33] Oquendo MA, Turret J, Grunebaum MF, Burke AK, Poh E, Stevenson E, Mann JJ, Galfalvy H, 2013. Sex differences in clinical predictors of depression: a prospective study. J. Affect. Disord 150, 1179–1183. 23735213 10.1016/j.jad.2013.05.010 PMC3759613

[R34] Perry VH, Nicoll JA, Holmes C, 2010. Microglia in neurodegenerative disease. Nat. Rev. Neurol 6, 193–201. 20234358 10.1038/nrneurol.2010.17

[R35] Pesarico AP, Chagas PM, Nacher J, 2021. Editorial: Animal Models of Stress - Current Knowledge and Potential Directions. Front. Behav. Neurosci 15, 655214. 33664658 10.3389/fnbeh.2021.655214 PMC7920965

[R36] Rahimian R, Belliveau C, Chen R, Mechawar N, 2022. Microglial Inflammatory-Metabolic Pathways and Their Potential Therapeutic Implication in Major Depressive Disorder. Front. Psych 13, 871997. 10.3389/fpsyt.2022.871997 PMC9245023 35782423

[R37] Rubinow DR, Schmidt PJ, 2019. Sex differences and the neurobiology of affective disorders. Neuropsychopharmacology 44, 111–128. 30061743 10.1038/s41386-018-0148-z PMC6235863

[R38] Sahasrabuddhe V, Ghosh HS, 2022. Cx3Cr1-Cre induction leads to microglial activation and IFN-1 signaling caused by DNA damage in early postnatal brain. Cell Rep. 38, 110252. 35045285 10.1016/j.celrep.2021.110252

[R39] Schramm E, Waisman A, 2022. Microglia as Central Protagonists in the Chronic Stress Response. Neurol Neuroimmunol Neuroinflamm 9. 10.1212/NXI.0000000000200023 PMC9453699 36357946

[R40] Seedat S, Scott KM, Angermeyer MC, Berglund P, Bromet EJ, Brugha TS, Demyttenaere K, de Girolamo G, Haro JM, Jin R, Karam EG, Kovess-Masfety V, Levinson D, Medina Mora ME, Ono Y, Ormel J, Pennell BE, Posada-Villa J, Sampson NA, Williams D, Kessler RC, 2009. Cross-national associations between gender and mental disorders in the World Health Organization World Mental Health Surveys. Arch. Gen. Psychiatry 66, 785–795. 19581570 10.1001/archgenpsychiatry.2009.36 PMC2810067

[R41] Seibenhener ML, Wooten MC, 2015. Use of the Open Field Maze to measure locomotor and anxiety-like behavior in mice. J Vis Exp, e52434. 25742564 10.3791/52434 PMC4354627

[R42] Sequeira-Cordero A, Salas-Bastos A, Fornaguera J, Brenes JC, 2019. Behavioural characterisation of chronic unpredictable stress based on ethologically relevant paradigms in rats. Sci. Rep 9, 17403. 31758000 10.1038/s41598-019-53624-1 PMC6874551

[R43] Shors TJ, Millon EM, Chang HY, Olson RL, Alderman BL, 2017. Do sex differences in rumination explain sex differences in depression? J. Neurosci. Res 95, 711–718. 27870434 10.1002/jnr.23976

[R44] Tsyglakova M, Huskey AM, Hurst EH, Telep NM, Wilding MC, Babington ME, Rainville JR, Hodes GE, 2021. Sex and region-specific effects of variable stress on microglia morphology. Brain Behav Immun Health 18, 100378. 34820640 10.1016/j.bbih.2021.100378 PMC8600001

[R45] Walsh JG, Muruve DA, Power C, 2014. Inflammasomes in the CNS. Nat. Rev. Neurosci 15, 84–97. 24399084 10.1038/nrn3638

[R46] Wang H, He Y, Sun Z, Ren S, Liu M, Wang G, Yang J, 2022. Microglia in depression: an overview of microglia in the pathogenesis and treatment of depression. J. Neuroinflammation 19, 132. 35668399 10.1186/s12974-022-02492-0 PMC9168645

[R47] Weissman MM, Bland RC, Canino GJ, Faravelli C, Greenwald S, Hwu HG, Joyce PR, Karam EG, Lee CK, Lellouch J, Lepine JP, Newman SC, Rubio-Stipec M, Wells JE, Wickramaratne PJ, Wittchen H, Yeh EK, 1996. Cross-national epidemiology of major depression and bipolar disorder. J. Am. Med. Assoc 276, 293–299. 8656541

[R48] Westfall S, Caracci F, Estill M, Frolinger T, Shen L, Pasinetti GM, 2021. Chronic Stress-Induced Depression and Anxiety Priming Modulated by Gut-Brain-Axis Immunity. Front. Immunol 12, 670500. 34248950 10.3389/fimmu.2021.670500 PMC8264434

[R49] Wohleb ES, Franklin T, Iwata M, Duman RS, 2016. Integrating neuroimmune systems in the neurobiology of depression. Nat. Rev. Neurosci 17, 497–511. 27277867 10.1038/nrn.2016.69

[R50] Wohleb ES, Terwilliger R, Duman CH, Duman RS, 2018. Stress-Induced Neuronal Colony Stimulating Factor 1 Provokes Microglia-Mediated Neuronal Remodeling and Depressive-like Behavior. Biol. Psychiatry 83, 38–49. 28697890 10.1016/j.biopsych.2017.05.026 PMC6506225

[R51] Woodburn SC, Bollinger JL, Wohleb ES, 2021. The semantics of microglia activation: neuroinflammation, homeostasis, and stress. J. Neuroinflammation 18, 258. 34742308 10.1186/s12974-021-02309-6 PMC8571840

[R52] Yankelevitch-Yahav R, Franko M, Huly A, Doron R, 2015. The forced swim test as a model of depressive-like behavior. J Vis Exp. 10.3791/52587 PMC4401172 25867960

