# Supplementary figures and images for: The role of the Toll like receptor 4 signaling in sex-specific persistency of depression-like behavior in response to chronic stress
Source: Brain Behav Immun. Author manuscript; Available in PMC 2024 Jun 3. (PMC11146676; doi:10.1016/j.bbi.2023.10.006)

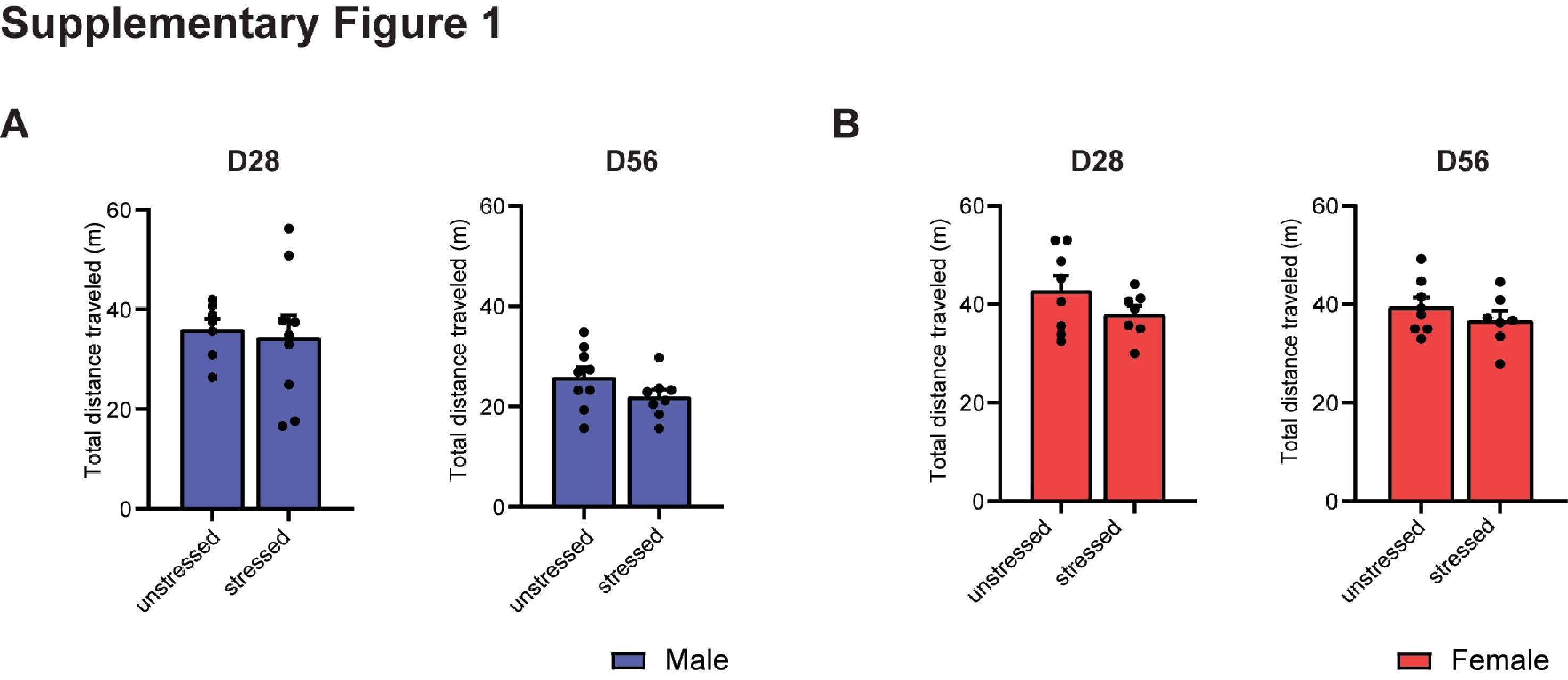

Supplement: Supple 1 [file NIHMS1990043-supplement-Supple_1.jpg]

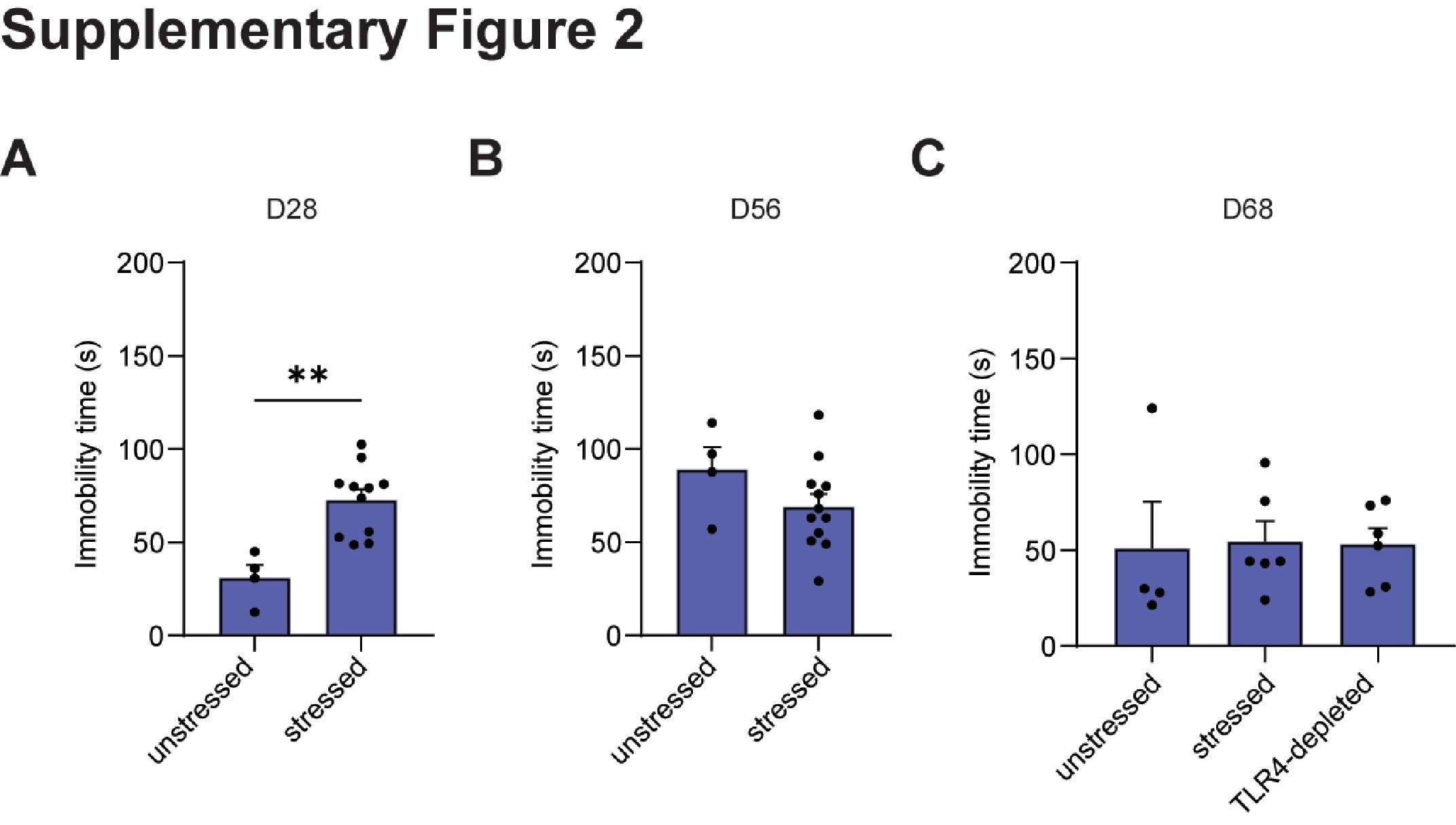

Supplement: Supple 2 [file NIHMS1990043-supplement-Supple_2.jpg]

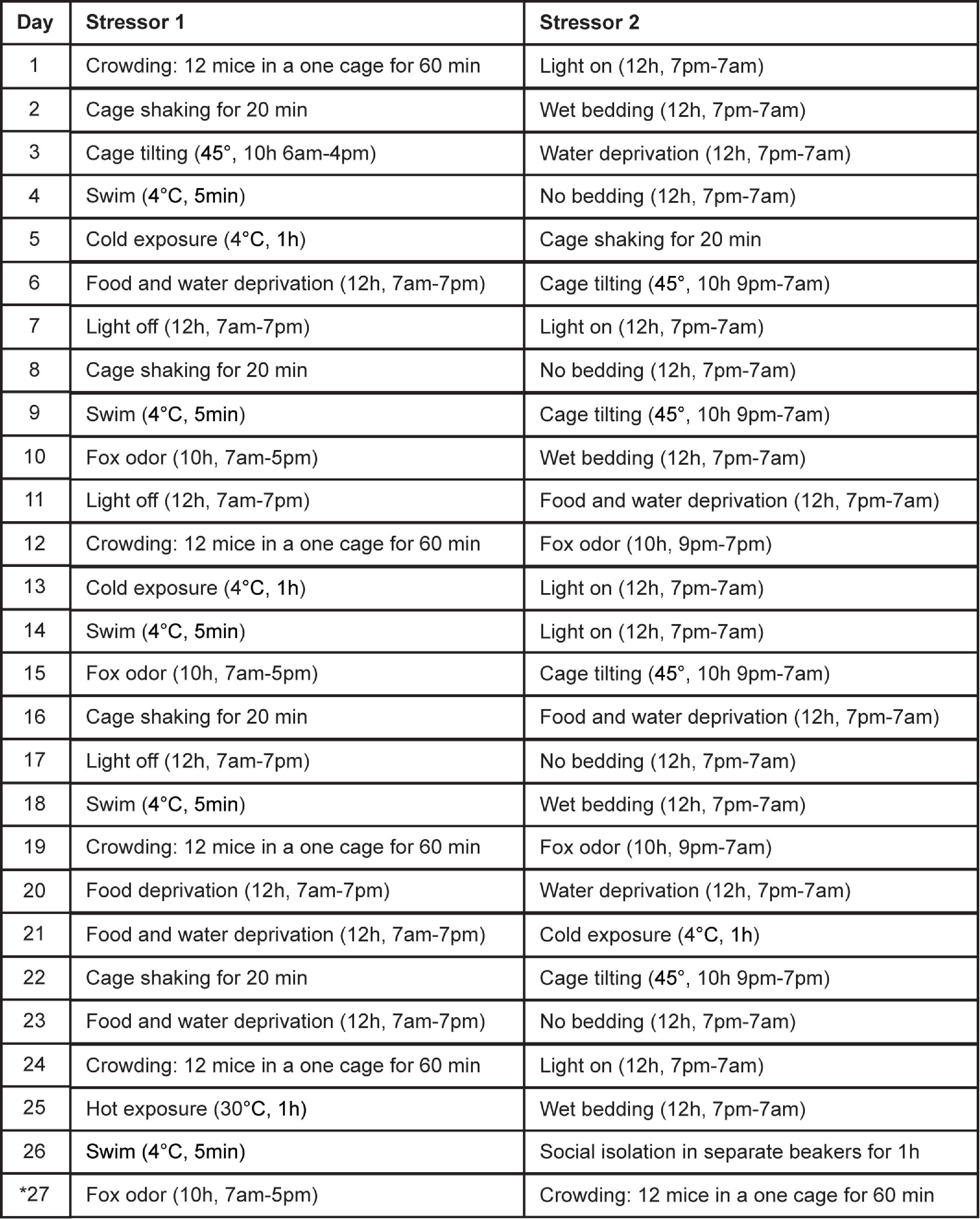

Supplement: Supple 3 [file NIHMS1990043-supplement-Supple_3.jpg]
